# Challenging the Illusion: Health Equity Amidst New Variants

**DOI:** 10.3389/ijph.2022.1604896

**Published:** 2022-05-02

**Authors:** Mohammad Yasir Essar, Arush Lal, Shoaib Ahmad, Faisal A. Nawaz, Salah Eddine O. Kacimi, Jaffer Shah, Ahsan Zil-E-Ali, Rajeev K. Singla, Atanas G. Atanasov, Bairong Shen

**Affiliations:** ^1^ Clinical Informatics Research Unit, Faculty of Medicine, University of Southampton, Southampton, United Kingdom; ^2^ Kabul University of Medical Sciences, Kabul, Afghanistan; ^3^ Department of Health Policy, London School of Economics and Political Science, London, United Kingdom; ^4^ Women in Global Health, Washington, DC, United States; ^5^ Community & Civil Society Representative, ACT Accelerator, London, United Kingdom; ^6^ Department of Medicine, District Headquarters Hospital, Faisalabad, Pakistan; ^7^ College of Medicine, Mohammed Bin Rashid University of Medicine and Health Sciences, Dubai, United Arab Emirates; ^8^ Department of Medicine, Faculty of Medicine, University of Tlemcen, Tlemcen, Algeria; ^9^ Medical Research Center, Kateb University, Kabul, Afghanistan; ^10^ Heart and Vascular Institute, Pennsylvania State University, Hershey, PA, United States; ^11^ Institutes for Systems Genetics, Frontiers Science Center for Disease-Related Molecular Network, West China Hospital, Sichuan University, Chengdu, China; ^12^ iGlobal Research and Publishing Foundation, New Delhi, India; ^13^ Ludwig Boltzmann Institute for Digital Health and Patient Safety, Medical University of Vienna, Vienna, Austria; ^14^ Institute of Genetics and Animal Biotechnology, Polish Academy of Sciences, Magdalenka, Poland

**Keywords:** vaccination, health equity, health disparities, COVID-19, variants

## Highlights

• LMICs with limited capacities and infrastructures have experienced striking and disproportionate public health and economic losses during the COVID-19 pandemic—particularly due to imposed lockdowns and restrictions.
• The pandemic’s emerging variants are a manifestation of unequal and unjust distribution of COVID-19 vaccination—unmasking “health equity” as an illusion.
• No firm actions have been taken by HICs and powerful actors, who could be playing a leading role in offering solutions rather than privileging self-defeating interests.
• The ongoing COVID-19 response and future efforts for pandemic preparedness should ensure health equity is made an urgent, core priority—rather than an afterthought.

When the first cases of COVID-19 emerged, the world had appeared unified in finding solutions to control the pandemic and mitigate its impacts. After several months of research, scientists rapidly collaborated to develop lifesaving medical countermeasures, like vaccines. It was considered a breakthrough in the pandemic, leading many to believe the end of the crisis was within reach. Over 2 years since cases were first diagnosed, the death toll has reached almost six million and is still climbing [1]. Despite over a year of calling for vaccine equity, much work remains.

During the course of the crisis, different trends of the COVID-19 pandemic have been observed globally. Countries with a declining GDP were facing recession and had a higher mortality rate, suggesting that economic status may be a useful predictor for COVID-19 related mortality, which has been disproportionately recorded worldwide [2]. In particular, low- and middle-income countries (LMICs) have experienced undulating waves of new cases and deaths throughout the pandemic. These fluctuations enabled the emergence of new (often more transmissible) variants, leading to a higher incidence of the disease in these countries and pushing their health system capacity to its limits [3]. It was observed that in many LMICs, there was a lack of available hospital beds, reduced ventilatory support, and a lack of available health care workers to treat critical cases [4]. This burden can be directly traced to numerous socioeconomic factors, including a lack of equitable distribution of vaccines across the world, managerial incompetency in vaccine delivery, limited public health experts to support risk mitigation strategies, chronic austerity for health system financing, and inadequate resources to maintain nationwide campaigns against COVID-19.

In addition to affecting public health, the pandemic has taken a significant toll on the economies of LMICs. Notably, the rapid spread of COVID-19 exacerbated unemployment rates and placed already-vulnerable populations at greater risk of poverty—fueling a dangerous cycle of impoverishment and ill health [5]. Despite several lockdown recommendations to ensure social distancing and control disease transmission, many communities in LMICs had no choice but to forgo these restrictions to continue seeking employment to meet their financial needs [6]. The lack of social protections and welfare to support these populations led to the continued spread of the virus.

While the challenge of enforcing non-pharmaceutical interventions has proven particularly difficult in LMICs, because of the heightened economic pressures, people have been forced to continue work and not comply with preventive measures [7]. This was compounded by unscientific, and unjust travel bans levied on several LMICs [8]. Many experts contend these practices did little to stop international transmission while enacting significant economic disruptions to impacted countries, exacerbating the strain on community health workers and local health systems.

As we enter year three of the pandemic and contend with Omicron as the latest variant, one might ask: Why haven’t we marked the end of the pandemic yet? And why there is such a huge disparity in vaccination across the globe? The answer to these questions hinges on our understanding of health equity.

Equity is one of the defining aspects of the Sustainable Development Goals (SDGs), and its lessons for the current COVID-19 pandemic have taken center stage in response and recovery efforts. Health equity is defined as the opportunity to seek good and fairer healthcare services without any barriers [9]. Rather than catalyzing on the emergence of the pandemic as an opportunity to unite behind bold proposals to support gender equity and vulnerable communities that were at most risk, all levels of health systems—from local to national—showed an alarming propensity to sideline already-marginalized populations and prioritize care for those in power or those who could afford it [10]. This scenario was also reflected globally, with LMICs often last in line to obtain vaccines and other critical COVID-19 tools.

Recent variants like Omicron are a direct consequence of the striking disparity between high-income countries (HICs) and LMICs in the context of health equity [11]. HICs, with greater access to financial resources, have in many ways continued to adopt measures that might prolong the pandemic, including prioritizing mass vaccination to their populations while pushing LMICs further back in the queue [12]. With each subsequent (booster) dose, LMICs have been left out. Moreover, HICs have simultaneously blocked manufacturers’ ability in LMICs from producing vaccines themselves, forcing these countries to rely on last-minute donations. LMICs had to heavily rely on COVAX and other bilateral donation programs, which have resulted in fewer doses of the vaccines. To compound this, the price of vaccine doses is also not feasible for LMICs to afford, with some reports of vaccine manufacturers even increasing the costs of doses for LMICs—a move that is ultimately self-defeating for global public health [13].

Omicron is just the latest in a series of alarm bells that the pandemic is still not under control. The virus will continue to circulate wherever immunization coverage is poor—often in LMICs. In countries where the variant is confirmed, travel restrictions have been imposed. This has caused a massive economic blow for LMICs, given that travel and international trade is considered a highly profitable sector.

The continued delays in vaccine delivery to LMICs causes the doses to get closer to expiry dates, forcing many recipient countries to reject them because of inability to distribute on time. This has facilitated the conditions for the virus to mutate and continue circulating, while placing LMIC recipient countries in a difficult position. The creation of vaccine manufacturing hubs in different regions and intellectual property (IP) waivers offer concrete solutions to curb the pandemic faster and prevent future crises by placing the power of disease mitigation in the hands of LMICs; however, this requires major private sector companies to prioritize human life over profit—a principle many have failed to achieve.

Finally, while major initiatives have been launched to strengthen global health security, including the pandemic treaty, IHR negotiations, a pandemic preparedness fund, ACT-Accelerator, and more, all of these mechanisms have largely continued to sideline the outsized role that routine health systems play in mitigating public health threats. Such initiatives must be developed with a view to strengthening health systems toward universal health coverage, maintaining essential health services during crises, protecting and supporting a trained community health workforce, and financing multisectoral and diverse country-led teams (with strong civil society and community representation) that can be rapidly mobilized to coordinate a robust response. Key to this is ensuring that health equity is not an afterthought, but rather a calculated and proactive approach to strengthening health security.

In a world fueled by health inequities and steeped in the harmful legacies of colonial and neoliberal pressures, coupled with unequal distribution of the COVID-19 vaccines, has paved the way for harmful variants to emerge. What is known as “health equity” has been unmasked as nothing more than an illusion. No firm actions have been taken by HICs and powerful actors who could be playing a leading role in offering solutions rather than privileging self-defeating interests. If these unfair and unjust practices continue, we are unlikely to see the end of the pandemic any time soon. HICs and major global health donors have an important role to play in righting these wrongs. The time to commit to unified, coordinated solutions is now.

## References

[1] COVID Live - Coronavirus Statistics - Worldometer (2022). Available from: https://www.worldometers.info/coronavirus/ (Accessed Jan 27, 2022).

[2] Bank for international Settlements (2020). Available from: https://www.bis.org/publ/work910.pdf (Accessed Jan 27, 2022).

[3] United Nation. COVID Cases Surging in Africa at Fastest Rate This Year, but Deaths Remain Low. United Nation: UN News Global Perspective Human Stories (2021). Available from: https://news.un.org/en/story/2021/12/1107882 .

[4] Pasquale S Gregorio GL Caterina A Francesco C Beatrice PM Vincenzo P COVID-19 in Low- and Middle-Income Countries (LMICs): A Narrative Review from Prevention to Vaccination Strategy. Vaccines (2021) 9:1477. 10.3390/VACCINES9121477 34960223 PMC8704834

[5] Global Humanitarian Response Plan COVID-19 (2021). Available from: https://www.unocha.org/sites/unocha/files/Global-Humanitarian-Response-Plan-COVID-19.pdf (Accessed March 14, 2022).

[6] Shadmi E Chen Y Dourado I Faran-Perach I Furler J Hangoma P Health Equity and COVID-19: Global Perspectives. Int J Equity Health (2020) 19:104–16. 10.1186/S12939-020-01218-Z/METRICS 32586388 PMC7316580

[7] Chowdhury R Luhar S Khan N Choudhury SR Matin I Franco OH. Long-Term Strategies to Control COVID-19 in Low and Middle-Income Countries: An Options Overview of Community-Based, Non-pharmacological Interventions. Eur J Epidemiol (2020) 35:743–8. 10.1007/S10654-020-00660-1 32656618 PMC7354877

[8] World Closing Its Doors to African Countries Due to Omicron (2021). Available from: https://www.aa.com.tr/en/americas/world-closing-its-doors-to-african-countries-due-to-omicron/2434131 (Accessed Feb 1, 2022).

[9] Social Determinants of Health (2022). Available from: https://www.who.int/westernpacific/health-topics/social-determinants-of-health .

[10] COVID-19 Pandemic Demonstrates Multilateral Cooperation Key to Overcoming Global Challenges. President Stresses as General Assembly Concludes Annual Debate | Meetings Coverage and Press Releases [Internet]. [Cited 2022 Mar 14]. Available from: https://www.un.org/press/en/2020/ga12273.doc.htm .

[11] The Lancet Infectious Diseases. Emerging SARS-CoV-2 Variants: Shooting the Messenger. Lancet Infect Dis (2022) 22:1. 10.1016/S1473-3099(21)00770-2 34895554 PMC8660034

[12] Geiger S McMahon A. Analysis of the Institutional Landscape and Proliferation of Proposals for Global Vaccine Equity for COVID-19: Too Many Cooks or Too Many Recipes? J Med Ethics (2021) 0:medethics–2021. 10.1136/MEDETHICS-2021-107684 34848492

[13] Rackimuthu S Narain K Lal A Nawaz FA Mohanan P Essar MY Redressing COVID-19 Vaccine Inequity amidst Booster Doses: Charting a Bold Path for Global Health Solidarity, Together. Glob Health (2022) 18(1):23. 10.1186/s12992-022-00817-5 PMC8862404 35193616

